# Effects of extreme temperatures on cardiovascular emergency hospitalizations in a Mediterranean region: a self-controlled case series study

**DOI:** 10.1186/s12940-017-0238-0

**Published:** 2017-04-04

**Authors:** Anna Ponjoan, Jordi Blanch, Lia Alves-Cabratosa, Ruth Martí-Lluch, Marc Comas-Cufí, Dídac Parramon, María del Mar Garcia-Gil, Rafel Ramos, Irene Petersen

**Affiliations:** 1 Vascular Health Research Group (ISV-Girona), Institut Universitari d’Investigació en Atenció Primària Jordi Gol (IDIAP Jordi Gol), c/ Maluquer Salvador, 11 baixos, Girona, 17002 Catalonia Spain; 2 Girona Biomedical Research Institute (IDIBGi), c/ del Dr. Castany, s/n, Salt, Girona, 17190 Catalonia Spain; 3 Universitat Autònoma de Barcelona, Cerdanyola del Vallès, Bellaterra Spain; 4 Centre d’Atenció Primària Santa Clara, Gerència d’Àmbit d’Atenció Primària Girona, Institut Català de la Salut, Girona, Spain; 5 Department of Medical Sciences, School of Medicine, Campus Salut, University of Girona, Girona, Spain; 6 Department of Primary Care and Population Health, University College of London, London, UK; 7 Department of Clinical Epidemiology, Aarhus University, Aarhus, Denmark

**Keywords:** Cold snap, Heat, Coronary heart disease, Stroke, Heart failure, Climate change, Hospital admissions, Weather

## Abstract

**Background:**

Cold spells and heatwaves increase mortality. However little is known about the effect of heatwaves or cold spells on cardiovascular morbidity. This study aims to assess the effect of cold spells and heatwaves on cardiovascular diseases in a Mediterranean region (Catalonia, Southern Europe).

**Methods:**

We conducted a population-based retrospective study. Data were obtained from the System for the Development of Research in Primary Care and from the Catalan Meteorological Service. The outcome was first emergency hospitalizations due to coronary heart disease, stroke, or heart failure. Exposures were: cold spells; cold spells and 3 or 7 subsequent days; and heatwaves. Incidence rate ratios (IRR) and 95% confidence intervals were calculated using the self-controlled case series method. We accounted for age, time trends, and air pollutants; results were shown by age groups, gender or cardiovascular event type.

**Results:**

There were 22,611 cardiovascular hospitalizations in winter and 17,017 in summer between 2006 and 2013. The overall incidence of cardiovascular hospitalizations significantly increased during cold spells (IRR = 1.120; CI 95%: 1.10–1.30) and the effect was even stronger in the 7 days subsequent to the cold spell (IRR = 1.29; CI 95%: 1.22–1.36). Conversely, cardiovascular hospitalizations did not increase during heatwaves, neither in the overall nor in the stratified analysis.

**Conclusions:**

Cold spells but not heatwaves, increased the incidence of emergency cardiovascular hospitalizations in Catalonia. The effect of cold spells was greater when including the 7 subsequent days. Such knowledge might be useful to develop strategies to reduce the impact of extreme temperature episodes on human health.

**Electronic supplementary material:**

The online version of this article (doi:10.1186/s12940-017-0238-0) contains supplementary material, which is available to authorized users.

## Background

Extreme outdoor temperatures have been reported to increase mortality in many parts of the world [1–3]. For example, a heatwave caused 70,000 deaths in Europe in 2003 [4], and a cold spell caused 140,000 deaths in China in 2008 [5]. Outdoor temperatures may also worsen many conditions, such as respiratory, infectious or cardiovascular diseases [6–8]. Although cardiovascular disease are one of the main causes of mortality worldwide, the effect of extreme outdoor temperature on cardiovascular diseases remains less evident [9].

Previous studies assessing the effect of hot temperatures and heatwaves on cardiovascular morbidity are scarce and dissonant. Some reviews showed a weak effect of hot temperatures on cardiovascular diseases in the elderly [6], and in general population [10], but such effect was not found in a meta-analysis by Turner et al. 2012 [9]. Another meta-analysis by Phung et al. aligned [11] with Turner et al. regarding the lack of effect of hot temperatures, but reported a positive association of heatwaves with cardiovascular hospitalizations.

Extreme cold increases the risk of cardiovascular hospitalization not only during cold spells but also some days after the cold spell episode occurred [7–11]. Studies assessing the impact of cold spells on health have mainly been conducted in cold-weather regions of Russia [16], Canada [12], China [13], USA [14] and United Kingdom [15]. However, the adverse health effects of cold temperatures might be more pronounced in warmer climates [8] because both people and housing conditions are not well adapted to such conditions. Therefore it is necessary to assess the effect of cold spells in regions with mild winters, such is the case for the Mediterranean region.

The Mediterranean region is a good place to study the effect of extreme weather on health due to its climatic and sociocultural characteristics. The Mediterranean region is especially vulnerable to global warming: since the 1960s, it has become warmer [17] and an increment of the frequency, intensity, and duration of heatwaves has been forecasted [18]. The impact of heat on human health might be reinforced by the “urban heat effect”: Mediterranean cities tend to be compact and densely populated what might induce a strong “urban heat effect” [17]. Therefore a thorough understanding of the effects of extreme outdoor temperature on human health is crucial to design targeted public health interventions that mitigate both the social and economic burden associated to cardiovascular diseases.

In this study we aimed to assess the effect of both cold spells and heatwaves on emergency hospitalizations due to cardiovascular diseases in Catalonia, a Mediterranean region from Southern Europe.

## Methods

### Study design

We conducted a population-based retrospective study. We used a self-controlled case series methodology (SCCS) [19, 20], to compare hospitalization rates for cardiovascular diseases during periods of exposure to extreme temperatures with rates during non-exposure periods within the same individual. The SCCS methodology relies on inferences based on within-person comparisons; thus, the potential confounding effect of individual characteristics that remain constant over time–such as gender and ethnicity–is cancelled out [19, 20].

### Data sources

Hospitalization data were obtained from the System for the Development of Research in Primary Care (SIDIAP) [21, 22]. The SIDIAP is a valid and reliable database for biomedical research which contains anonymized, longitudinal medical records of 6 million patients (10% of the Spanish population and 80% of the Catalan population) attended by the Catalan Public Health Service (Institut Català de la Salut, ICS). SIDIAP comprises electronic medical records, including comprehensive demographic information, clinical diagnoses, referral and hospitalizations. The high quality of these data has been previously documented [21, 22], specifically for cardiovascular risk factors and diseases [23, 24].

Meteorological data were obtained from the Catalan Meteorological Service. We used data from 161 automatic weather stations spread out throughout Catalonia that measure environmental temperature (C°) every 1 hour. We estimated the exposure to outdoor temperature for each individual by linking their census tracks (registered in SIDIAP) to the closest weather station (in distance). In our study, distance between subjects’ home and the closest automatic weather station was in average 3.9 km (standard deviation, SD = 2.5 km). This estimate is commonly used in epidemiological studies when dealing with the effect of outdoor temperature on health [25], and it has been reported that ambient temperature recorded at the nearest weather station may be associated with personal heat exposure [26].

Pollution data were obtained from 43 automatic stations from the Network for Monitoring and Forecasting of Air Pollution from the Catalan Ministry of Territory and Sustainability. Previous studies showed that exposure to air pollution might contribute to develop cardiovascular disease [27]. We obtained composite data that provides a daily estimation of the Catalan Air Quality Index (CAQI). This index weights the contribution of the levels of carbon monoxide, nitrogen dioxide, sulphur dioxide, ozone and particle matter (PM10) in order to indicate the air quality using the following categories: very poor (CAQI < −50), poor (−50 ≤ CAQI < 0), low (0 ≤ CAQI < 25), acceptable (25 ≤ CAQI < 50), satisfactory (50 ≤ CAQI < 75) or excellent (75 ≤ CAQI < 100). The median distance between subjects’ home and the closest automatic station was 15.6 km (SD = 9.8 km).

### Setting

Catalonia is a region (32.000 km^2^) of Spain located in the western Mediterranean basin (latitude 41°23’N, longitude 2°9’E). The climate is predominantly Mediterranean with 4 season year, relatively mild winters and hot and dry summers.

### Follow-up

We included data from 2006 to 2013. The observation period comprised January and February when studying cold spells, and July and August when studying heatwaves. These are the coldest and hottest periods of the year respectively, when the vast majority of coldspells and heatwaves occur. Furthermore, restricting the study time to 2 months periods favours one of the assumptions of the self-control case series methodology: constant event rates within study intervals of time [19]. Censoring was applied at the earliest of the two following events: death, transfer from SIDIAP, dismantlement of the closest weather or pollution station or end of the study period.

### Study population

We included individuals aged 18 years or over inhabiting in Catalonia who had an emergency hospitalization due to cardiovascular diseases. We considered cardiovascular events occurred in January or February to assess the effect of cold spells, and those occurred in July or August to study heatwaves. To restrict to first episodes of cardiovascular disease, we excluded individuals with evidence of previous history of stroke, coronary heart disease, heart failure or transient ischemic attack.

### Outcome and exposures

The study outcome was first emergency hospitalizations due to cardiovascular disease (including coronary heart disease, stroke or heart failure) of each individual in the study population (ICD-9 and ICD-10 codes are shown in Additional file 1).

We defined cold spell as a period of at least 3-day length with daily minimum temperatures lower than the 5^th^ percentile of the daily minimum temperature. Percentiles were estimated using data of January and February from 2006 until 2013 for each weather station. We defined heatwave as a period of at least 3-day length with daily maximum temperatures higher than the 95^th^ percentile of the daily maximum temperature in a given weather station for July and August in a similar manner. The definition of cold spells or heatwaves based on percentiles is common in studies dealing with the effect of environmental temperature on health [11]. The biggest effect for extreme heat has been reported to occurred immediately [28, 29], while for extreme cold the effect might last until several days after the cold spells [11, 30]. Therefore, we considered one exposure to assess extreme heat (days during heatwaves), and three different exposures when studying extreme cold weather: cold spells; the accumulative 3-day effect of cold spells, i.e. a period that includes the cold spell and the subsequent 3 days; and the accumulative 7-day effect of cold spells, i.e. a period that includes the cold spell and the subsequent 7 days [11].

### Statistical analyses

We used the self-controlled case series (SCCS) methodology [23] to assess the relative incidence rate ratios (IRRs) of hospitalizations during periods of extreme temperature exposure in comparison to reference time periods with normal temperature exposure. Since comparisons are made within individuals, the SCCS relies on conditional Poisson regression to calculate IRR and 95% confidence intervals (CIs). We estimated the IRR for cardiovascular events for each exposure period compared with non-exposure period, adjusting by age categories (18–35; 36–55; 56–75; >75 years old), time intervals—fortnights-, and daily exposure to air pollution (very poor; poor; low; acceptable; satisfactory; or excellent air quality). We assumed the following multiplicative model as the incidence function:

$$ {\uplambda}_{i jklm} = \exp \left({\varPhi}_i + {\upalpha}_j + {\upgamma}_k + {\updelta}_l + {\upbeta}_m\right) $$

where φ_*i*_ representes an effect for each individual *i*, α_*j*_ represents an age group effect, γ_*k*_ represents the forthnights effect, δ_*l*_ represents the air pollution exposure effect, and β_*m*_ represents the cold spell or heatwave exposure effect. Additional stratified analyses were undertaken by type of cardiovascular disease (heart failure, coronary heart disease or stroke), by age categories (<65 years; ≥65 years), and by gender, since we wanted to examine if the effects of extreme temperatures were different in these groups.

When using the SCCS method some assumptions are required to provide unbiased estimates [19]. First, the SCCS is suitable for independent recurrent events; since cardiovascular events are not independent—having one cardiovascular event increases the risk of the next cardiovascular events—we considered only the first event for each individual. Second, bias may be introduced if the outcome influences the likelihood of future exposures; in our study, the onset of cardiovascular events was totally unrelated to a future exposure to extreme outdoor temperatures. Thus, we allowed for repeated exposures, that is, individuals could experience several heatwaves or cold spells through the observation period. Third, event rates are assumed to be constant within the time intervals; to test if this assumption was violated, we performed sensitive analyses by dividing the observation period in fortnights and adding this term to the model. Fourth, bias may also arise if the outcome events lead to censoring, preventing future exposure assessment; in our study cardiovascular death could prevent future exposures to some individuals, therefore we performed a sensitivity analysis excluding individuals whose follow-up ended within 90 days of their cardiovascular event [31].

All data analysis was conducted using the Stata version 13.

## Results

During January and February from 2006 to 2013, there were 22,611 emergency hospitalizations due to cardiovascular diseases. The mean age at study entry was 71.9 years (sd = 13.4 years), 10,726 (47%) were women, 14,273 (63%) had one single cardiovascular event and 7,687 (34%) died before the study end (28 February 2013). Median follow-up was 415 days (Inter Quartile Range: 296 to 474 days), what equals to 7 winters—considering only January and February. In average the daily minimum temperature was 1.4 °C ± 4.6 °C during the study period and dropped to −6.7 °C ± 4.6 °C during cold spells. The cold spell median threshold, i.e. the 5^th^ percentile of the minimum daily temperature in each weather station, was −4.1 °C (Inter Quartile Range: −6 °C to −2 °C). Annual descriptives of temperature, cold spells occurrence and duration, and frequency of emergency hospitalizations due to cardiovascular diseases are shown in Table 1.

**Table 1:** Descriptives of temperature, extreme temperature episodes (cold spells or heatwaves) and emergency cardiovascular hospitalizations by year

	2006	2007	2008	2009	2010	2011	2012	2013
Temperature (°C)								
Winter^a^ T_min_	1.1	2.9	3.2	1.7	1.3	1.7	−0.2	1.4
Summer^a^ T_max_	28.9	27.3	27.8	29.0	28.8	27.5	28.9	28.4
Cold spells								
Number^b^	1 (0–1)	30 (0–1)	0 (0–0)	1 (0–1)	77 (0–2)	135 (0–1)	207 (0–3)	16 (0–1)
Duration^c^ (days)	3 (3–3)	4 (3–5)	–	3 (3–3)	4 (3–10)	5 (3–6)	6 (3–12)	4 (3–5)
CV hospitalizations^d^	1	6	–	16	64	251	410	0
Heatwaves								
Number^b^	39 (0–2)	20 (0–1)	9 (0–1)	52 (0–1)	6 (0–2)	50 (0–1)	152 (0–2)	22 (0–1)
Duration^c^ (days)	4 (3–11)	3 (3–4)	3 (3–6)	4 (3–8)	4 (3–7)	3 (3–4)	6 (3–11)	3 (3–3)
CV hospitalizations^d^	41	9	24	69	1	19	178	4

^a^Average of the daily minimum temperature (T_min_) during January and February or daily maximum temperature (T_max_) during July and August in overall Catalonia

^b^Sum (range per weather station) of the number of extreme temperature episodes (ETEs) detected in the 161 weather stations

^c^Mean (range) of the ETEs overall duration detected in each weather station

^d^Total number of first emergency cardiovascular hospitalizations occurred during ETEs

**Fig. 1:**
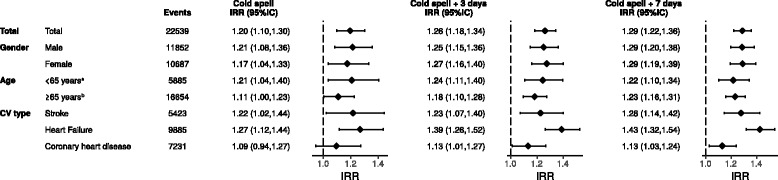
Incident rate ratios (*IRR*) for cardiovascular emergency hospitalization considering three exposures: cold spells, cold spells and the next 3 days, and cold spells and the next 7 days. All models were adjusted by age, fortnight and air pollution. Results are shown stratified by gender, age groups and cardiovascular (*CV*) event type. ^*a*^Age groups used for adjustment in models restricted to population < 65 years old: 18–35, 36–55; 56–64. ^*b*^Age groups used for adjustment in models restricted to population ≥65 years old; 65–70, 71–75, 76–80, >80

The incidence of emergency hospitalizations due to cardiovascular disease increased significantly during cold spells compared with unexposed periods (IRR = 1.20; CI 95%: 1.10–1.30) (Fig. 1). Cold spells significantly increased the risk to be hospitalized due to cardiovascular disease in both sexes in a similar manner (Fig. 1). The effect was similar in people younger than 65 years (IRR = 1.21; CI 95%: 1.04–1.40) and older people (IRR = 1.11; CI 95%: 1.00–1.23). The impact of cold spells differed slightly between cardiovascular type events. The effect of cold spells was significantly associated with stroke (IRR = 1.23; CI 95%: 1.02–1.44) and heart failure (IRR = 1.27; CI 95%: 1.12–1.44), but not with coronary heart disease (IRR = 1.10; CI 95%: 0.94–1.27). Sensitivity analysis showed similar results (Additional file 2).

When accounting for the 3-day accumulative effect of cold spells, i.e. including a ‘lag period’ of the subsequent 3 days, we found an increased effect (IRR = 1.26; CI 95%: 1.18–1.34) compared with the unexposed period. The accumulative effect remains up to 7 days after the cold spell. When we accounted for the 7-day accumulative effect of cold spells, we observed an increased effect (IRR = 1.29; CI 95%: 1.22–1.36) compared with the unexposed period. In both accumulative periods (3 or 7 days), similar estimates were observed for sex and age groups (Fig. 1), and slightly higher estimates were found for heart failure in comparison with other individual outcomes (Fig. 1). Further adjustments or the exclusion of cardiovascular deaths did not alter the results (Additional file 2).

During July and August from 2006 to 2013, 17,017 emergency hospitalizations due to cardiovascular diseases registered by SIDIAP fulfilled the inclusion criteria. The mean age at study entry was 70.1 years (sd = 13.9), 7,615 (45%) were women, 11,508 (68%) had one single cardiovascular event and 5,244 (31%) died before the conclusion of the study (31 August 2013). Median follow-up was 434 days (Inter Quartile Range: 310 to 496 days), what equals to 7 summers—considering only July and August. On average, the daily maximum temperature was 28.1 °C ± 5.3 °C during the study period and raised to 33.8 °C ± 4.9 °C during heatwaves. The median of the heatwave threshold, i.e. the 95^th^ percentile of the maximum daily temperature in each weather station, was 34 °C (Inter Quartile Range: 32 °C to 35.6 °C). Annual descriptives of temperature, heatwaves occurrence and duration, and frequency of emergency hospitalizations due to cardiovascular diseases are shown in Table 1.

**Fig. 2:**
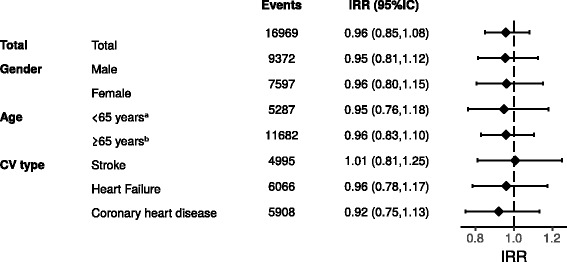
Incident rate ratios (*IRR*) for cardiovascular emergency hospitalization during heatwaves. All models were adjusted by age, fortnight and air pollution. Results are shown stratified by gender, age groups and cardiovascular (*CV*) event type. ^*a*^Age groups used for adjustment in models restricted to population < 65 years old: 18–35, 36–55; 56–64. ^*b*^Age groups used for adjustment in models restricted to population ≥65 years old; 65–70, 71–75, 76–80, >80

The effect of heatwaves on overall cardiovascular hospitalizations was not significantly different from the null (Fig. 2). No significant differences were observed when stratifying by sex, age or cardiovascular type categories (Fig. 2). Finally, sensitivity analyses did not modify the study findings (Additional file 3).

## Discussion

This study offers a comprehensive examination of the effect of extreme atmospheric temperatures over the risk of emergency cardiovascular hospitalizations, by assessing the impact of cold spells and heatwaves. The novelty of this study is to provide a broad perspective not only by assessing the impact of extreme atmospheric temperature in a a Mediterranean region but also by reporting results stratified by gender, age and type of cardiovascular event, in order to enhance comparability with other studies. To our knowledge, this is the first study dealing with the effects of cold spells on cardiovascular morbidity in a region with Mediterranean climate, which is characterized by mild winter and hot and dry summers.

We found that cold spells but not heatwaves increased emergency hospitalizations due to cardiovascular diseases in Catalonia. The results of our study are in line with Ryti et al. 2016, who suggested that the adverse health effects of cold temperatures might be more pronounced in warmer climates due to worse adapted housing to cold weather. This might also be the case in the studied area, where housing insulation is stated to need improvement [32], and where about 5.2% of Catalan population in 2013 suffered energy poverty, they could not afford proper household heating [33]. The effect of temperature on health may be relative to the prevailing climate rather than associated with a universal threshold of absolute temperature [8].

We found an incidence increase of cardiovascular hospitalizations of 20% during cold spells. Significant associations between cold spells and cardiovascular hospitalizations were previously reported in other parts of the world: in China cold spells were associated with a 33% (95% CI: 28, 37%) increase of cardiovascular hospital admissions [13]; in the USA with a 36% increase [14]; and in Russia the number of strokes doubled during cold spells [16].

Several plausible mechanisms are potentially involved in increasing the risk of cardiovascular hospitalizations due to extreme cold exposure. Low outdoor temperature might induce vasoconstriction and might increase systolic and diastolic blood pressure [34], blood viscosity, blood cholesterol, platelet count, and red blood cell count, which may increase the risk of atheromatous plaque rupture, myocardial infarction, or stroke [8].

We observed a significant association between cold spells and cardiovascular emergency hospitalizations in all stratified analyses, except for coronary heart diseases. All sensitivity analyses pointed out that the risk of coronary heart disease did not increase during cold spells. These results are in line with previous studies showing that myocardial infarction or ischaemic heart disease were little or not affected by weather [35, 36]. However, other studies pointed out a significant association between temperature and mortality due to acute myocardial infarction [37, 38].

We observed an increment of the incidence of cardiovascular hospitalizations of 29% during cold spells and the following 7 days. There is general consensus about the lagged effect of cold spells on cardiovascular morbidity. For example, in the United Kingdom the risk of cardiovascular hospitalization increased more than two-fold during cold spells together with a 3-day lagged period [15]; and in the Czech Republic the excess cardiovascular mortality persisted longer with cold spells than with heatwaves [37]. A weaker effect of the cold spell compared with lagged period was also detected in studies assessing the association between moderate decrease in atmospheric temperature—not necessarily a cold spell—and cardiovascular morbidity [39–41] or cardiovascular mortality [30, 42].

Mechanisms explaining the lagged effect of cold spells on cardiovascular morbidity remains poorly understood. Carder et al. 2005 suggested that the lag in the effects of low temperature on human health may vary in length for different adverse health outcomes: vasoconstriction and blood pressure changes in seconds or minutes, while concentrations of fibrinogen increase in hours. Therefore consequences such as myocardial ischaemia from increased work demands on the heart muscle or from increased formation of thrombus can ensue in hours or days [42].

Our results pointed out stronger lagged effects in all stratified analyses, particularly in elderly and for heart failure. In relation to the elderly, our findings are in agreement with previous studies that suggested that cold-related cardiovascular mortality for people aged over 60 years peaked several days after younger people did [37, 43]. In relation to heart failure, we detected that the effect tended to be stronger in the lag period (IRR = 1.43; 95% CI: 1.32–1.54) than for cold spell effects (IRR = 1.27; 95% CI: 1.12–1.44). This finding is consistent with a study from Canada that showed stronger effects of cold weather on heart failure mortality for lagged exposure rather than the exposure at the same day when the cold weather occurred [44].

We did not observe any association between heatwaves and risk of cardiovascular emergency hospitalizations. Our findings are in line with those observed in previous reviews: Turner et al. 2012 performed a meta-analysis showing a lack of association between increased outdoor temperature and cardiovascular morbidity [9]; whilst Bashakaran et al. 2012 showed inconclusive results. However, other authors found a positive association between the risk of cardiovascular hospitalizations and heatwaves [11].

The lack of association between cardiovascular morbidity and heatwaves can be partially explained by the fact that cardiovascular risk factors tended to be lower in the summer [34, 45]. The impact of heatwaves, which occurred in a short period of time, may not be strong enough to counteract the protective effect of lower levels of cardiovascular risk factors in the summer. Other factors could also contribute to explain the lack of association between heatwaves and cardiovascular morbidity. One of them could be the immediate impact of heatwaves on mortality: vulnerable people might die before being admitted into the hospital, resulting in a drop of cardiovascular hospitalizations [9, 46, 47]. Another factor could be that vulnerable people might have intentionally avoided outdoor exposures during extreme hot weather [11] encouraged by the action plan to prevent the impact of heatwaves implemented by the regional government [48, 49]. Our results did not show any association between outdoor extremely high temperature and cardiovascular hospitalizations in any of the stratified analyses. Our results indicated there was no effect of heatwaves either in men or women, in line with previous literature [50–52]. The possible gender differences regarding the increase of cardiovascular risk associated to heatwaves have been poorly studied; therefore, our study provides new insights that might be compared with future studies. Our results indicated a similar effect of heatwaves in age-groups. This finding differed from previous studies which suggested that the elderly were more vulnerable to heatwaves than younger people [37, 53]. However, recent research showed that the effect of extreme heat on the elderly might differ depending on the disease: extreme heat was associated with increased hospital admissions for renal or respiratory diseases, but not for cardiovascular diseases amongst the elderly [54]; other studies found a positive association only for heat stroke but not for other cardiovascular outcomes in people aged 65 years or older [28]. Therefore, the evidence that the elderly would be the highest-risk age group for heatwaves-related illness is not evident regarding cardiovascular morbidity.

Our study has several strengths. First, accesss to validated, high-quality, electronic medical records [22] provided a large sample size and large number of cardiovascular events, reflected real-life conditions, and warranted high external validity. Moreover, the large sample size allowed us to constrict the study period avoiding periods with higher rate of cardiovascular hospitalizations, such as Christmas [55], and thus avoiding violation of the SCCS assumption of constant events along time. Second, by using the self-controlled case series design, we automatically controlled for all time-fixed individual level factors that could be related to the association between outdoor temperature and human health, such as socioeconomic status [56] or body fat [26]. Third, we adjusted the models by air pollution which was previously reported as an inducing factor to develop cardiovascular diseases [57] or mortality [58], although we did not find an effect of pollution on cardiovascular disease. Fourth, we used percentiles to define heat or cold spells which are a commonly used criteria [11], making our study easily comparable. Finally, this study contributes to understand the effect of both heatwaves and cold spells in an area with mild winters that has been scarcely addressed previously, the Mediterranean region.

We also acknowledge several limitations. First, one assumption of the self-controlled case series is that the occurrence of an event does not alter the likelihood of exposure; when this assumption is violated immortal bias occurs, resulting in overestimated IRR. We may have violated this assumption, since the occurrence of a cardiovascular event increases the risk of death. However, we provided sensitivity analyses that reassured the results. Second, our data did not include potentially important factors related to exposure to atmospheric temperature such as time spent outdoors, housing isolation, use of heating or cooling systems, which have been proven to significantly reduce the effect of temperature on heat-related hospitalizations [52]. Third, it has been suggested that in-hospital excess of cardiovascular deaths were a masked comorbid condition rather than the primary diagnosis responsible for hospitalization [59]; we could no discard misclassification in our dataset.

## Conclusions

Cold spells but not heatwaves increased emergency hospitalizations due to cardiovascular diseases from 2006 through 2013 in a Mediterranean region (Catalonia, Southern Europe). The impact of cold spells showed a stronger effect when the exposure encompassed the duration of the cold spell and a lagged period of 3 or 7 days. Moreover, we observed different impacts depending on the cardiovascular outcome, since coronary heart disease did not significantly increase emergency hospitalization during cold spells. Our findings might be helpful to encourage the development of a Cold Health Action Plan, to complement the already existing Heatwaves Action Plan [48] implemented by the regional government in Catalonia.

## Additional files

Additional file 1: Classification of Diseases codes (ICD, Ninth and Tenth revision). Description of data: ICD-9 and ICD10 used to define the study outcome. (DOCX 13 kb)

Additional file 2: Sensitivity analyses to assess the effect of cold spells on cardiovascular hospitalizations. Description of data: Incident rate ratios (IRR) for cardiovascular emergency hospitalization considering three exposures: cold spells, cold spells and the next 3 days, and cold spells and the next 7 days. Models adjusted by age, age and forthnight, and survivor analyses are shown. Results are shown stratified by gender, age groups and cardiovascular (CV) event type. ^a^Age groups used for adjustment in models restricted to population <65 years old: 18–35, 36–55; 56–64. ^b^Age groups used for adjustment in models restricted to population ≥65 years old; 65–70, 71–75, 76–80, >80. ^c^ Survivor Analyses: individuals whose follow-up ended within 90 days of their cardiovascular event were excluded. (PDF 37 kb)

Additional file 3: Sensitivity analyses to assess the effect of heatwaves on cardiovascular hospitalizations. Description of data: Incident rate ratios (IRR) for cardiovascular emergency hospitalization considering heatwaves as exposure. Models adjusted by age, age and forthnight, and survivor analyses are shown. Results are shown stratified by gender, age groups and cardiovascular (CV) event type. (PDF 32 kb)
