# Supplementary material for: Effects of extreme temperatures on cardiovascular emergency hospitalizations in a Mediterranean region: a self-controlled case series study
Source: Environ Health. 2017 Apr 4;16:32. doi: 10.1186/s12940-017-0238-0 (PMC5379535; doi:10.1186/s12940-017-0238-0)
Supplement: Supplementary file 1 — Classification of Diseases codes (ICD, Ninth and Tenth revision). Description of data: ICD-9 and ICD10 used to define the study outcome. (DOCX 13 kb) [file 12940_2017_238_MOESM1_ESM.docx]

We considered someone was hospitalized due to cardiovascular disease if the primary diagnosis registered in the hospital admission record was any one of the following Classification of Diseases codes (ICD, Ninth and Tenth revision): 410 and subcategories, 411.1, 411.8, 413 and subcategories, 414 and subcategories; I21 and subcategories, I20 and subcategories, I25 and subcategories for coronary heart disease; 434 and subcategories, 436 and subcategories, 437.0, 437.1, 438 and subcategories; I63 and subcategories, I66 and subcategories, I67 and subcategories, I69 and subcategories, for stroke; 428 and subcategories; I50 and subcategories, for heart failure.
