# Supplementary figures and images for: Effects of extreme temperatures on cardiovascular emergency hospitalizations in a Mediterranean region: a self-controlled case series study
Source: Environ Health. 2017 Apr 4;16:32. doi: 10.1186/s12940-017-0238-0 (PMC5379535; doi:10.1186/s12940-017-0238-0)

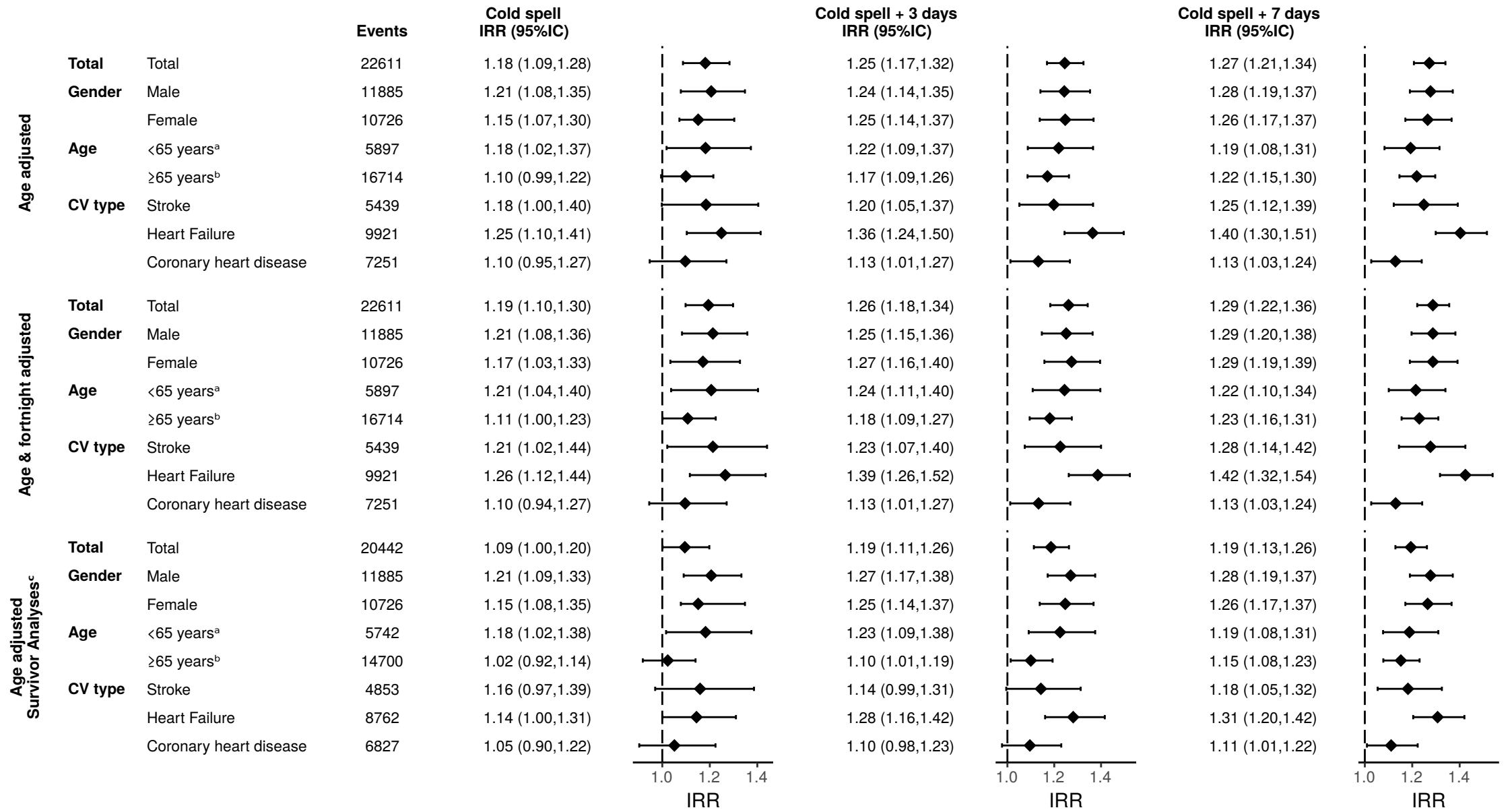

Supplement: Supplementary file 2 — Sensitivity analyses to assess the effect of cold spells on cardiovascular hospitalizations. Description of data: Incident rate ratios (IRR) for cardiovascular emergency hospitalization considering three exposures: cold spells, cold spells and the next 3 days, and cold spells and the next 7 days. Models adjusted by age, age and forthnight, and survivor analyses are shown. Results are shown stratified by gender, age groups and cardiovascular (CV) event type. aAge groups used for adjustment in models restricted to population <65 years old: 18–35, 36–55; 56–64. bAge groups used for adjustment in models restricted to population ≥65 years old; 65–70, 71–75, 76–80, >80. c Survivor Analyses: individuals whose follow-up ended within 90 days of their cardiovascular event were excluded. (PDF 37 kb) [file 12940_2017_238_MOESM2_ESM.pdf]

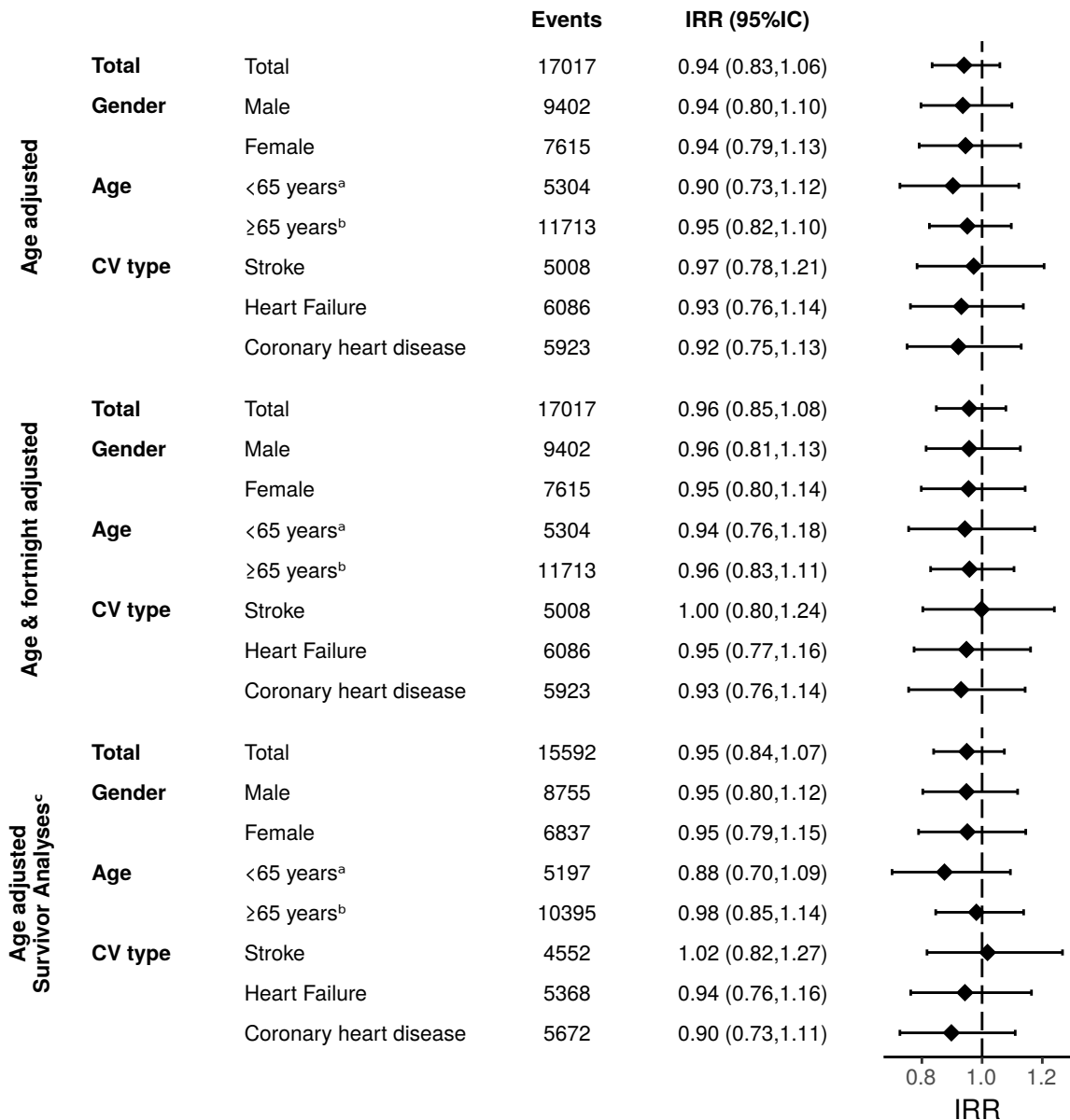

Supplement: Supplementary file 3 — Sensitivity analyses to assess the effect of heatwaves on cardiovascular hospitalizations. Description of data: Incident rate ratios (IRR) for cardiovascular emergency hospitalization considering heatwaves as exposure. Models adjusted by age, age and forthnight, and survivor analyses are shown. Results are shown stratified by gender, age groups and cardiovascular (CV) event type. (PDF 32 kb) [file 12940_2017_238_MOESM3_ESM.pdf]
